# Human Hepatocellular response in Cholestatic Liver Diseases

**DOI:** 10.1080/15476278.2023.2247576

**Published:** 2023-08-20

**Authors:** Kimberly Ortiz, Zeliha Cetin, Yiyue Sun, Zhiping Hu, Takeshi Kurihara, Edgar N. Tafaleng, Rodrigo M. Florentino, Alina Ostrowska, Alejandro Soto-Gutierrez, Lanuza A.P. Faccioli

**Affiliations:** a Department of Pathology, University of Pittsburgh School of Medicine, Pittsburgh, Pennsylvania, USA; b Pittsburgh Liver Research Center, Human Synthetic Liver Biology Core, University of Pittsburgh, Pittsburgh, Pennsylvania, USA; c University of Pittsburgh Drug Discovery Institute, University of Pittsburgh, Pittsburgh, Pennsylvania, USA; d McGowan Institute for Regenerative Medicine, Pittsburgh, Pennsylvania, USA

**Keywords:** Cholestasis, hepatocellular response, human hepatocytes, primary biliary cholangitis, primary sclerosing cholangitis

## Abstract

Primary biliary cholangitis (PBC) and primary sclerosing cholangitis (PSC), the most common types of cholestatic liver disease (CLD), result in enterohepatic obstruction, bile acid accumulation, and hepatotoxicity. The mechanisms by which hepatocytes respond to and cope with CLD remain largely unexplored. This study includes the characterization of hepatocytes isolated from explanted livers of patients with PBC and PSC. We examined the expression of hepatocyte-specific genes, intracellular bile acid (BA) levels, and oxidative stress in primary-human-hepatocytes (PHHs) isolated from explanted livers of patients with PBC and PSC and compared them with control normal human hepatocytes. Our findings provide valuable initial insights into the hepatocellular response to cholestasis in CLD and help support the use of PHHs as an experimental tool for these diseases.

## GRAPHICAL ABSTRACT

**Figure:**
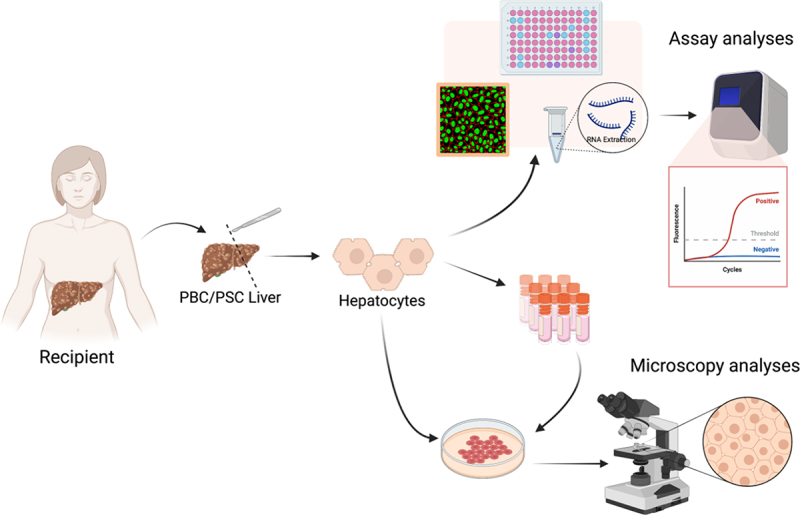

## Introduction

Cholestatic liver diseases (CLDs) are a group of disorders characterized by impaired bile formation and flow.^1^ This disruption of bile flow from the liver to the small intestine leads to the accumulation of toxic bile acids and bilirubin, oxidative stress, and, finally, hepatocellular injury, often culminating in liver failure and the need for transplantation for these patients.^1,2^ CLDs are responsible for nearly 10% of liver transplants in the United States^3^ and despite recent advances in the understanding of the underlying mechanisms of CLDs, there is still a significant gap in therapeutic options for these conditions.^4^ Two major types of CLDs are primary biliary cholangitis (PBC) and primary sclerosing cholangitis (PSC). Both PBC and PSC are known to have a strong genetic basis, with environmental factors also contributing to disease development.^2,4–6^

The prevalence of PBC and PSC ranges from 1.91 to 40.2 per 100,000 and one to 16 per 100,000, respectively, with PBC having a strong female predominance.^2,7^ PBC is an autoimmune disease characterized by destructive lymphocytic cholangitis and the presence of anti-mitochondrial antibodies.^4^ The pathophysiology of PBC involves loss of immune tolerance to biliary epithelial cells, leading to progressive fibrosing cholangitis, cholestasis, and resultant liver fibrosis.^2,4^ PSC is also caused by an immune-mediated attack on the biliary system and is characterized by biliary inflammation, periductal fibrosis, and cholestasis.^5,6^ While PBC primarily affects small intrahepatic ducts, PSC involves both intrahepatic and extrahepatic bile ducts.^2^

Although uncommon, these diseases cause considerable morbidity and mortality.^2,4–6^ Unfortunately, therapies for PBC and PSC are still lacking despite the numerous studies that have been conducted on cholestasis. Although PBC has few available treatments to help slow disease progression, such as Ursodeoxycholic Acid (UDCA), no effective medical treatment currently exists for PSC.^2,4^ Many patients will instead only receive symptomatic management until they are suffering from end-stage liver disease (ESLD) and eligible for liver transplantation.^2^ The largely unknown etiology and disease mechanisms of PBC and PSC have made therapeutic research challenging.^2^

Human cholangiocytes from patients with PBC and PSC have recently been characterized and studied ex vivo in few published reports.^8–10^ These CLD cholangiocytes were found to have increased expressions of senescence and inflammatory markers, as well as inhibited cell growth and proliferation, and these cellular characteristics were found to be associated with clinical disease severity and prognosis.^8–10^ However, the hepatocellular response to the chronic cholestatic states of these CLDs has not yet been thoroughly investigated. Hepatocytes are central to the pathogenesis of CLDs and are the primary site of BA synthesis, metabolism, and transport. It is therefore essential to study these hepatocytes for their reactive pathways and for potential therapeutic targets in CLD.

Although disease processes begin within cholangiocytes, the resulting CLD is secondary to hepatocellular oxidative stress and death.^2,4^ In cholestatic conditions, studies have suggested that hepatocytes may have an adaptive response involving upregulation of genes promoting β-oxidation of fatty acids, BA detoxification and excretion, and antioxidant defense and a downregulation of those responsible for BA synthesis and uptake.^11,12^ However, the ways in which these cholestatic feedback pathways may be affected by the disease mechanisms of PBC or PSC have not been well-established in human hepatocytes.

While much of the current literature focuses on the bile ducts, immune system, or gut-liver axis, we examined how hepatocytes respond and cope with the cholestatic injury from PBC and PSC. Therefore, the aims of this study are to characterize hepatocytes from livers of patients with PBC and PSC and begin our investigations on how hepatocytes respond to these cholestatic states.

## Material and methods

### Isolation of human hepatocytes

The University of Pittsburgh HRPO has determined that the human hepatocyte isolation protocol employed for the collection of samples from liver explants is exempt from further review (IRB STUDY20090069). PBC (*n* = 5) and PSC (*n* = 5) hepatocytes were prepared from a left lobular section of resected recipient livers during liver transplantation. Controls (*n* = 5) were obtained from resected portions of non-diseased human donor livers that were procured for liver transplantation, but not used clinically for medical reasons. Resected livers were protected from ischemic injury by flushing with ice-cold University of Wisconsin (Belzer) solution immediately after vascular clamping and resection in the Operating Room, then placed on ice and transported immediately to the cell isolation laboratory (Supplementary table 1).

Hepatocytes were isolated from encapsulated left lateral segment liver segments by a modified three-step perfusion technique.^13^ Briefly, the specimens were placed in the custom-made perfusion apparatus and two hepatic vessels were cannulated with tubing attached to a multi-channel manifold. Each catheter was secured in place by suturing or clamping adjacent tissue with a hemostat. Cell isolation was initiated via perfusion (recirculation technique) with calcium-free HBSS (cat. 14025126 Sigma Aldrich) supplemented with 1 ml of 0.5 mM EGTA (cat. NC0997810,Bio World) and then with EMEM (cat. BW12136Q, Lonza) supplemented with 1 ml of CaCL_2_ (cat. C7902, Sigma Aldrich) and with collagenase/protease (cat. 007–1010, VitaCyte, LLC) solution diluted with 5 and 3 ml of water, until the tissue was fully digested. Both the HBSS and EMEM solutions were warmed to 37°C in a water bath prior to starting. The digestion time for each preparation ranged from 30 to 60 min, depending mostly on the overall quality of the tissue.

The digested liver was removed, immediately cooled with ice-cold Leibovitz’s L-15 medium (cat. 21-083-027, GIBCO^TM^) supplemented with 5% of fetal bovine serum (cat. F4135, Sigma Aldrich), and filtered through a 215 and 125 µm mesh. The final crude cell suspensions were centrifuged three times at 600RPM for 7 min. We then separate the non-parenchymal cells using Percoll purification and microscopy evaluation, achieving approximately 97% of hepatocyte purity in the end of the protocol. The yield and viability of freshly isolated hepatocytes were estimated by trypan blue staining, and the cells were separated for culturing and cryopreservation.

### Cryopreservation of human hepatocytes

For cell cryopreservation, the Cryostor CS10 medium (Biolife Solutions) was used. Cell suspensions (7×10^6^ cells/ml of cryopreservation medium) were aliquoted into 2 ml cryotubes, stored in −80°C for 5 h, and then transferred to a liquid nitrogen tank for long-term storage.

### Hepatocyte culture

The cells were resuspended (0.75×106/ml) in complete HMM medium (Lonza) supplemented with 7% FBS and dispensed into 6-well plates (2 ml/well) pre-coated with collagen type I. After a 24-h incubation at 37°C in 5% CO_2_, the medium was changed to serum-free complete HMM and cell cultures were evaluated using EVOS M5000 (Invitrogen). The same culture process was executed for all cells recovered from cryopreservation.

Patient demographic data, as well as cell concentration, viability, and plating efficiency of freshly isolated and cryopreserved hepatocytes, are presented in Supplementary Table S1.

### Quantitative real time PCR

Total RNA was isolated from PBC and PSC cells using RNeasy Mini kits (QIAGEN, Hilden, Germany) and reverse transcribed using SuperScript III (Invitrogen) following the manufacturers’ instructions. We performed qPCR with a StepOnePlus system (Applied Biosystems) using TaqMan Fast Advanced Master Mix (Invitrogen). The probes used are listed in Supplementary Table 2. Relative gene expression was normalized to ACTB mRNA. Relative expression was calculated using the ΔΔCT method.

### Bile acid assay

Intracellular bile acid levels were measured using a Bile Acid Assay Kit (Sigma-Aldrich, MAK309) according to the manufacturer’s instructions. Cell suspensions (5×106 cells/mL) were lysed using a nonionic lysis buffer containing 1% Triton. Samples were then briefly homogenized via gentle pipetting and sonicated using a digital ultrasonic cleaner (Sentry) for 10 min. To ensure cell lysis, this process was repeated for a total of 20 min of sonication. Fluorescence intensities of samples at 585 nm were measured using a plate reader (BioTek Synergy HTX), and BA levels were calculated (Gen5).

### Immunostaining

Human hepatocytes were fixed with 4% PFA for 15 min, washed three times with PBS, and stored at −20°C. On the day of staining, samples were washed three times for 5 min with wash buffer (PBS, 0.1% BSA, and 0.1% TWEEN 20) before being blocked and permeabilized in blocking buffer (PBS, 10% normal donkey serum, 1% BSA, 0.1% TWEEN 20, and 0.1% Triton X-100) for an hour at room temperature. The cells were then incubated with anti-8-OHdG primary antibody (Novus Bio, NB600–1508-50ul, 1:400 in blocking buffer) overnight at 4°C. The following day, the cells were washed three times with wash buffer and incubated with secondary antibody (Invitrogen, Alexa flour 584, 1:250 in blocking buffer) for 2 h in the dark at room temperature. The samples were then washed three times with wash buffer and three times with PBS, counterstained with 1 µg/mL of DAPI (Sigma Aldrich) and finally mounted using Fluoromount-G with DAPI (Invitrogen) and cover glass. Samples were imaged using an Eclipse Ti inverted microscope (Nikon) and the NIS-Elements software platform (Nikon). Immunostaining was quantified using the intensity of red per area for the same number of randomly selected cells across the images of CLD (*n* = 4) and control samples (*n* = 3).

### Statistical methods

Data for qPCR and bile acid assay are presented as mean ± standard error of the mean (SEM), while data for categorical variables are presented as numbers and percentages. Since data for continuous variables were not normally distributed, p-values (P) were determined using unpaired two-tailed Mann-Whitney U tests with 95% confidence. For immunostaining data, p-values were determined using Welch’s test with a 95% CI. p-values ≤0.05 were considered statistically significant. Statistical analyses were performed using GraphPad Prism version 9.3.0.

## Results

**Figure 1.**
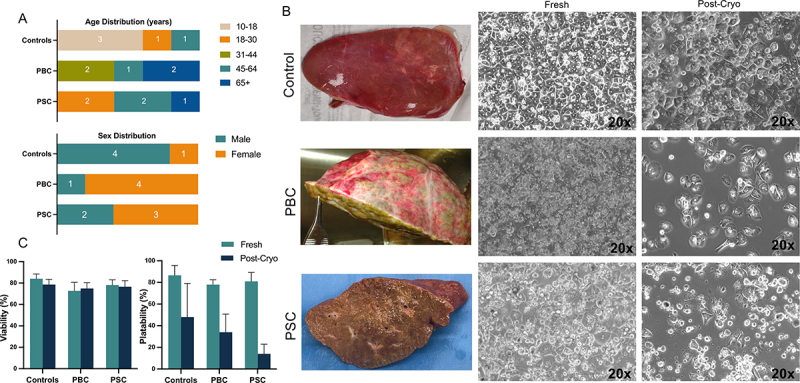
Patient demographics and sample imaging. (a) Demographic data showing sex and age distribution of patients (b) Example images of liver explant and phase-contrast photographs of cultured primary human hepatocytes, both 24-hours freshly isolated and post-cryo, of control, PBC, and PSC samples. (c) Viability and plating efficiency post-cryopreservation in controls, PBC and PSC hepatocytes.

PHHs were successfully isolated from five each of controls, PBC, and PSC livers. The control samples were obtained from patients who were markedly younger, with three pediatric patients at 10–18 years of age (Figure 1a). The patients with PBC and PSC included more females, four and three, respectively, than the control group (Figure 1a). The average viabilities of isolated hepatocytes were 72.8 ± 7.85% for PBC, 78.1 ± 4.73% for PSC, and 84 ± 4.53% for controls (Figure 1b, c). Plating efficiency, measured by percentage of cell adhesion, of freshly isolated hepatocytes from PBC and PSC livers after 24 h of culture was 78.0 ± 4.47% and 82.5 ± 8.66%, respectively (Figure 1b, c). After cryopreservation, the observed viability was similar, 75 ± 5.39% and 77 ± 6.48%, respectively (Figure 1b, c). However, plating efficiency of these cells was markedly reduced when cultured after cryopreservation, with an average of 34.0 ± 16.73% and 14.0 ± 8.94% (Figure 1b, c). Our controls showed a similar trend, with an average of 86.6 ± 9.10% plating efficiency before cryopreservation and 48 ± 31.1% after (Figure 1b, c).

**Figure 2.**
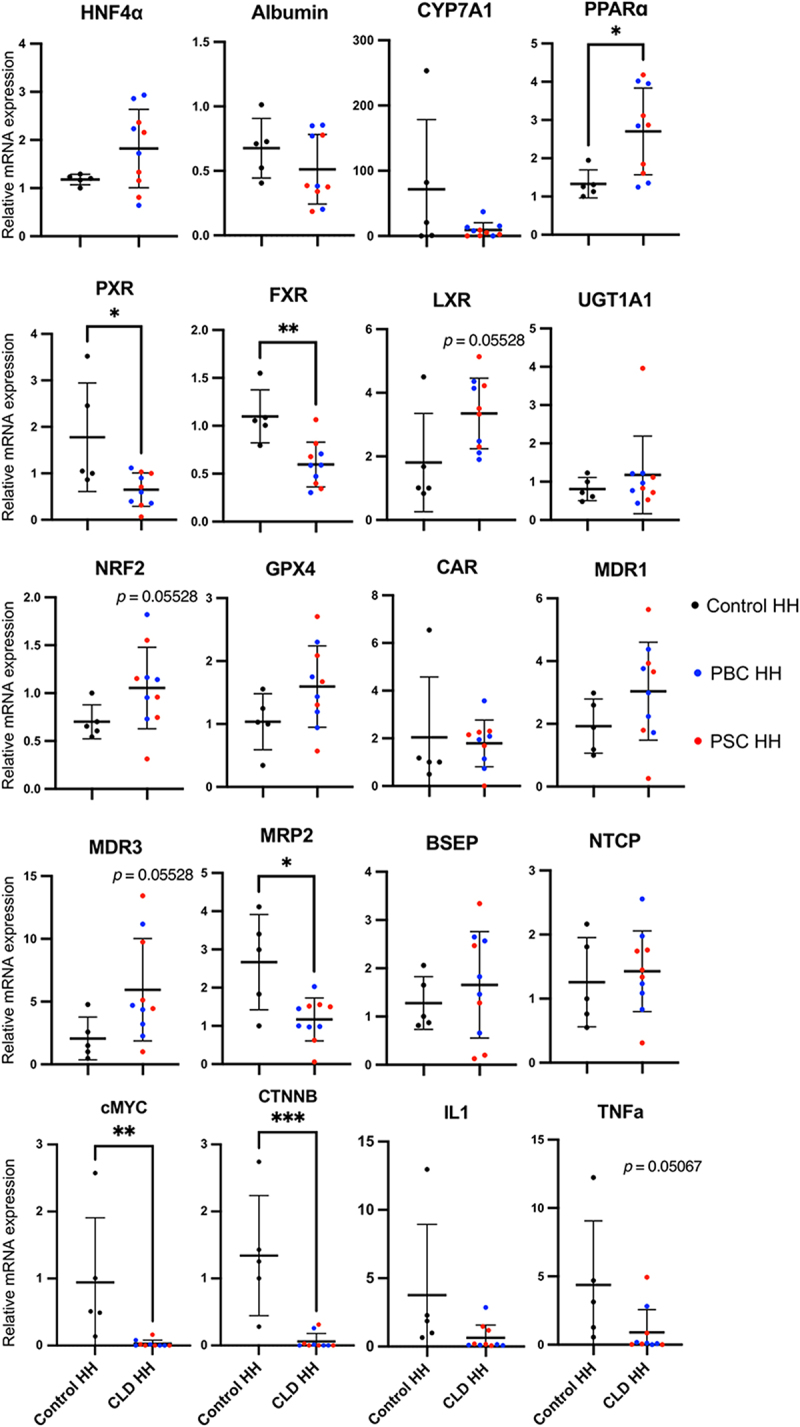
Primary human hepatocyte gene expression. mRNA analysis of the expression of HNF4α, Albumin, CYP7A1, PPARα, PXR, FXR, LXR, UGT1A1, NRF2, GPX4, CAR, MDR1, MDR3, MRP2, BSEP, NTCP, cMYC, CTNNB, IL1a and TNF. Values are determined relative to ACTB and presented as fold change relative to the expression in a consistent control sample, which is set as 1. Error bars represent mean ± SD of 5 samples for PBC (blue) and PSC (red), and Control livers (*p < 0.05, **p < 0.01).

These diseased hepatocytes also showed varied expressions for multiple genes related to bile acid transport and cellular response to cholestasis. The hepatocytes from CLD livers expressed significantly increased levels of PPARα (*p* = 0.0193), but decreased levels of FXR (*p* = 0.008), PXR (*p* = 0.0280), and MRP2 (*p* = 0.0193), when compared to controls (Figure 2). Genes related to cellular proliferation, cMYC (*p* = 0.0082) and CTNNB (*p* = 0.0005), were also significantly reduced in CLD samples. LXR (*p* = 0.5528), NRF2 (*p* = 0.05528), and MDR3 (*p* = 0.05528) exhibited a strong tendency of being increased in hepatocytes from CLD livers when compared to controls. Additionally, IL1a (*p* = 0.0785) and TNFα (*p* = 0.0507) exhibited a strong tendency of being decreased in our CLD samples (Figure 2).

**Figure 3.**
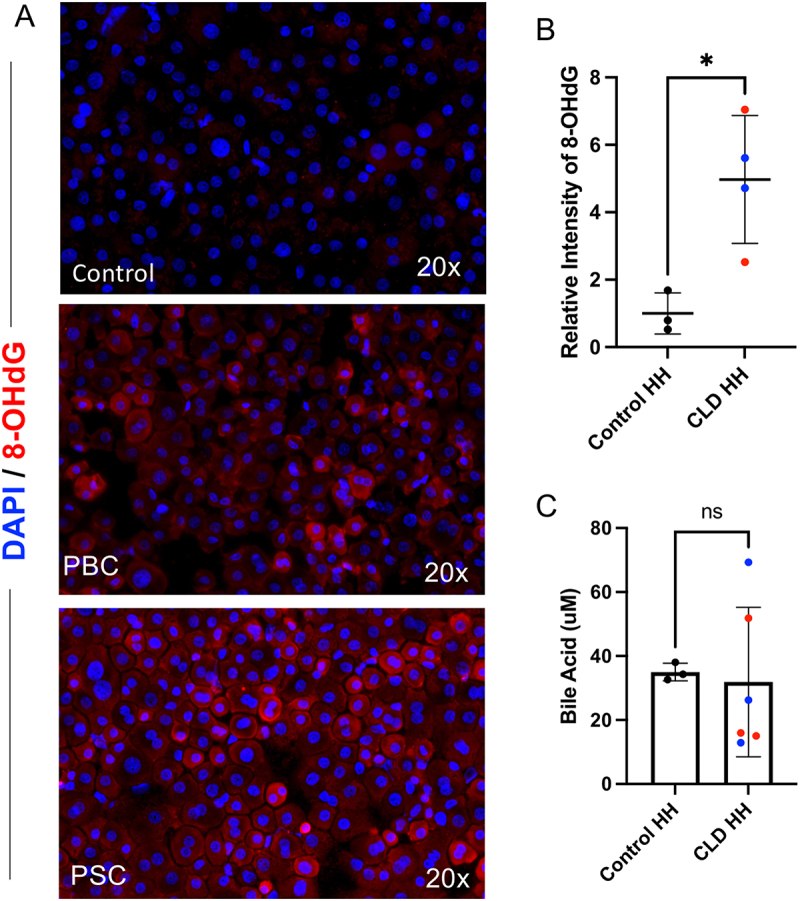
Oxidative stress and bile acid assay in primary human hepatocytes. (a) Immunofluorescence staining of fixed hepatocytes from a control liver and livers with PBC, showing 8-OHdG in red and cell nuclei in blue. (b) The relative intensity of these immunostainings is quantified in graph format with CLD HH samples (*n* = 4), including PBC (blue) and PSC (red), and Control HH samples (*n* = 3). (c) Bile Acid assay results are shown, with the CLD HH samples including PBC (blue) and PSC (red).

As a marker for oxidative stress, immunostaining for 8-hydroxydeoxyguanosine (8-OHdG) was performed on three control and four CLD hepatocyte samples (Figure 3a). Since 8-OHdG is a common reactive oxygen species-induced DNA lesion, it is widely considered as an index of DNA damage, for both nuclear and mitochondrial DNA.^14,15^ The CLD hepatocytes had a significantly higher relative fluorescence intensity of this marker when compared to control hepatocytes (*p* = 0.0191, 95% CI [1.099, 6.847]), suggesting increased levels of DNA damage due to oxidative stress within these cells (Figure 3b).

To assess bile acid accumulation in the CLD PHHs, intracellular BA levels were measured in three random samples from each of the groups. The results showed no significant difference between CLD and control hepatocytes, with the CLD samples having greater variation (Figure 3c). All values measured were within the assay’s linear detection range of 1–150 µM bile acids.

## Discussion

Hepatic response to cholestasis has predominantly been studied in various rodent models, with scarce data available from human samples.^16–19^ Furthermore, much of the literature using human specimens focuses on cholangiocytes or immune cells.^2^ Although these cells are important for the pathogenesis of CLDs, the resulting end-stage liver disease and failure are mainly secondary to hepatocyte death.^2, 4, 20^ The accumulation of toxic hydrophobic BAs that occurs during cholestasis is known to cause hepatocyte endoplasmic reticulum stress, mitochondrial damage, and release of inflammatory cytokines.^20–23^ Recent studies have also suggested that hepatocytes, not cholangiocytes or other non-parenchymal cells (NPCs), elicit the cytokine-induced inflammatory liver injury seen in CLDs.^18,21–23^ As researchers work to develop drugs for CLD targeting BA biosynthesis, metabolism, and excretion as well as liver inflammation and fibrosis, we decided to investigate the cells in which these processes occur: the human hepatocyte.

Our present study includes a characterization of hepatocytes isolated from explanted livers of patients with PBC and PSC. Our results suggest that PHHs, both freshly isolated and recovered from cryopreservation, represent a valuable research tool for evaluation of CLD. Although the plating efficiency was markedly reduced when culturing CLD PHHs after cryopreservation, this was observed in many of our control cells as well. This is consistent with previous studies showing that cryopreservation negatively affects attachment efficiency of hepatocytes, which may be due to loss of membrane integrity, a downregulation of attachment-related genes, and/or increased cryopreservation-induced apoptosis.^24,25^

To our knowledge, this is the first study that assessed the mRNA expression of BA-regulatory genes in PHHs isolated from human PBC and PSC livers. Most of the literature regarding hepatocellular response to cholestasis in PBC and PSC comes from animal models, many of which have contradictory findings.^4, 6, 11, 12^ Our results show that PPARα expression was significantly increased in CLD hepatocytes, suggesting an adaptive response to increase BA detoxification and β-oxidation of fatty acids during cholestasis.^2,17^ PPARα is also known to promote the expression of MDR3, which could explain the increased expression of MDR3 in our CLD hepatocytes, although this increase was not significant.^2,17^ Differences in a majority of genes were not significant, which might be due to the limited sample size in this study.

However, our qPCR results do not consistently support the expected increase in expression of genes responsible for BA detoxification and export and decrease in those responsible for BA import in CLD hepatocytes.^2,21^ Notably, the gene expression of the nuclear receptors FXR and PXR was significantly decreased and CYP7A1 did not significantly differ in CLD hepatocytes when compared to controls, which may instead support recent literature indicating dysfunctional FXR signaling and lack of CYP7A1 repression in both PBC and PSC.^11, 17, 19, 21, 26, 27^ These prior studies suggest that in advanced stages of PBC and PSC, there might not be as strong of an FXR response due to decreased BA signaling and/or a dysfunctional FXR-FGF19 gut – liver endocrine pathway.^11, 17, 19, 21, 26, 27^ Nevertheless, the disruption of these pathways during CLD remains controversial.^11, 17, 19, 21, 26, 27^

Genes related to cellular proliferation and inflammation were also assessed. CLD hepatocytes showed significantly decreased expression of genes involved in cellular proliferation, cMYC, and CTNNB. On the contrary, no significant differences were seen in expression of Inflammatory genes, IL1 and TNFα, when compared to controls. While we might have expected genes involving inflammation and hepatocyte proliferation to be upregulated during liver injury, it is important to note that the CLD samples in this study come from patients with decompensated ESLD, for which their liver function has been affected enough to require transplant surgery. These hepatocytes may therefore have different gene expression than what would be expected in early disease, as supported by published reports describing altered gene expression and immune dysfunction in advanced stages of liver disease.^19,28,29^ Additionally, there may also be an association with paradoxical expression of these genes and advanced disease progression, which we may be inadvertently selecting due to our samples being exclusively from end-stage, transplant patients with PBC and PSC.

Moreover, our study found that CLD hepatocytes exhibit increased oxidative stress, as assessed using 8-OHdG, a marker of oxidative DNA damage. Although the CLD results were more variable, likely due to the different stages of disease and decompensation in our patients, there was significantly more 8-OHdG found in these CLD samples. When staining, 8-OHdG was found mainly throughout the cytoplasm, demonstrating the abundance of oxidative mitochondrial damage in CLD hepatocytes.^14^ Gene expression for NRF2 and GPX4, which regulate antioxidant response, also tended to be higher in many of the CLD samples, although not significant, further suggesting a reactive response to the mitochondrial injury and oxidative stress in CLD.^4,17^ These results are consistent with published reports and support the role that oxidative stress has in the pathological mechanism of disease for PBC and PSC.^4,17^

The bile acid assay results showed no significant difference between bile acid concentrations in CLD and control hepatocytes. However, this assay only measures levels of non-sulfated bile acids. Sulfation of bile acids, catalyzed by SULT2A1, increases their solubility and decreases intestinal absorption, thus enhancing BA detoxification and excretion.^4,6^ Importantly, SULT2A1 expression is upregulated by PPARα, which was increased in our CLD samples.^4,6^ Therefore, these sulfated bile acids may be more numerous or variable in our CLD samples. It is also important to note that there is a wide range of decompensation in our CLD patients, which will also affect both sulfated and non-sulfated bile acid levels.

The present study provides valuable insights into the hepatic response to cholestasis in human CLD. Many of the genes characterized in this study are already being investigated as therapeutic targets for PBC and PSC. FXR, PXR, and PPAR agonists are also now being considered as anti-cholestatic agents.^2, 4, 18, 23, 26, 27^ Furthermore, the paradoxically reduced expressions of certain genes, such as those for FXR, PXR, and CTNNB, in CLD hepatocytes suggest that these cells may serve as an important target for therapeutic research investigating these genes.^2, 4, 17, 27, 30^ Additional promising therapeutic studies also focus on targeting the activation of hepatocyte detoxification enzymes, such as UGT1A1 and SULT2A1, to accelerate the metabolism of hydrophobic bile acids to hydrophilic bile acids, minimizing oxidative stress and protecting against hepatocellular injury.^1, 18, 31^

This study has some notable limitations, including restricted sample size due to disease scarcity and the need for further characterization in future studies. Nevertheless, this report is one of the very few that examine the hepatocytes and not cholangiocytes, in CLD. Our findings here provide valuable initial insights into the hepatocellular response to cholestasis in CLD and help support the use of PHHs as an experimental tool for these diseases. Although we acknowledge that the use of PHHs is limited in accessibility, their use provides valuable insight for studies using a variety of models for PBC and PSC. Still, the scarcity of experimental human models for PBC and PSC warrants further investigations into the pathological mechanisms of disease using PHHs. We expect that future therapeutic studies using human hepatocytes will prove to be essential to improving health outcomes for these patients.

## Conclusion

Human hepatocytes represent an important experimental tool that has been mainly overlooked in published reports regarding cholangiopathies. This study characterizes PHHs from patients with PBC and PSC and found that these hepatocytes express variable levels of genes vital to the pathogenesis of CLD and exhibit increased levels of oxidative stress. Thus, we provide valuable initial insights into the hepatocellular response to cholestasis in CLD. However, more studies characterizing PBC and PSC hepatocytes and their reactive pathways are needed to identify potential therapeutic targets for hepatocyte function maintenance in a cholestatic environment.

## Supplementary Material

Supplemental Material

## Abbreviations

CLD: Cholestatic Liver Disease
PBC: Primary Biliary Cholangitis
PSC: Primary Sclerosing Cholangitis
UDCA: Ursodeoxycholic Acid
BA: Bile Acid
ESLD: End-stage Liver Disease
PHH: Primary Human Hepatocyte
FBS: Fetal Bovine Serum
Npcs: Non-Parenchymal Cells
HNF4α: Hepatocyte Nuclear Factor 4 Alpha
CYP7A1: Cholesterol 7 Alpha-Hydroxylase
Pparα: Peroxisome Proliferator-Activated Receptor-Alpha
PXR: Pregnane X Receptor
FXR: Farnesoid X Receptor
LXR: Liver X Receptor
UGT1A1: UDP-Glucuronosyltransferase 1–1
NRF2: Nuclear Factor Erythroid 2–Related Factor 2
GPX4: Glutathione Peroxidase 4
CAR: Chimeric Antigen Receptor
MDR1: Multidrug Resistance 1
MDR3: Multidrug Resistance 3
MRP2: Multidrug Resistance-Associated Protein 2
BSEP: Bile Salt Export Pump
NTCP: Na^+^ -Taurocholate Cotransporting Polypeptide
