# Supplementary material for: Human Hepatocellular response in Cholestatic Liver Diseases
Source: Organogenesis. 2023 Aug 20;19(1):2247576. doi: 10.1080/15476278.2023.2247576 (PMC10444014; doi:10.1080/15476278.2023.2247576)
Supplement: Supplemental Material [file KOGG_A_2247576_SM6528.zip › Supplementary table 01.docx]

Table 1. Patient’s demographic data, yield and viability of isolated hepatocytes.

|  | AGE (YR) | SEX | DIAGNOSIS | CELL CONCENTRATION (106 /mL) | VIABILITY (%) | PLATABILITY (%) | VIABILITY POST-CRYO (%) | PLATABILITY POST-CRYO (%) |
| --- | --- | --- | --- | --- | --- | --- | --- | --- |
| 1 | 64 | F | PBC | 300 | 61 | 70 | 68 | 20 |
| 2 | 41 | F | PBC | 1600 | 76 | 80 | 80 | 60 |
| 3 | 65 | M | PBC | 600 | 75 | 80 | 80 | 20 |
| 4 | 68 | F | PBC | 428 | 82 | 80 | 76 | 40 |
| 5 | 44 | F | PBC | 691 | 70 | 80 | 71 | 30 |
| 6 | 23 | F | PSC | 1400 | 83 | 75 | 83 | 30 |
| 7 | 28 | M | PSC | 1000 | 82 | 85 | 82 | 10 |
| 8 | 66 | F | PSC | 640 | 80 | 90 | 70 | 10 |
| 9 | 55 | M | PSC | 792 | 73 | 70 | 73 | 10 |
| 10 | 62 | M | PSC | 394 | 73 | 85 | 75 | 10 |
| 11 | 11 | M | No CLD control | 2000 | 89 | 95 | 78 | 80 |
| 12 | 24 | F | No CLD control | 1000 | 78 | 80 | 73 | 10 |
| 13 | 16 | M | No CLD control | 9600 | 83 | 80 | 86 | 60 |
| 14 | 15 | M | No CLD control | 9400 | 88 | 98 | 80 | 70 |
| 15 | 60 | M | No CLD control | 100 | 82 | 80 | 76 | 20 |
