# Supplementary material for: Human Hepatocellular response in Cholestatic Liver Diseases
Source: Organogenesis. 2023 Aug 20;19(1):2247576. doi: 10.1080/15476278.2023.2247576 (PMC10444014; doi:10.1080/15476278.2023.2247576)
Supplement: Supplemental Material [file KOGG_A_2247576_SM6528.zip › Supplementary Table 02.docx]

|  | GENE NAME(S) | IDENTIFICATION CODE | COMPANY SOURCE |
| --- | --- | --- | --- |
| 1 | HNF4α | Hs00604431 | Thermo Fisher Scientific |
| 2 | Albumin | Hs00609411 | Thermo Fisher Scientific |
| 3 | CYP7A1 | Hs00167982 | Thermo Fisher Scientific |
| 4 | PPARα | Hs00947536 | Thermo Fisher Scientific |
| 5 | PXR (NR1I2) | Hs01114267 | Thermo Fisher Scientific |
| 6 | FXR (NR1H4) | Hs00231968 | Thermo Fisher Scientific |
| 7 | LXR (NR1H3) | Hs00172885 | Thermo Fisher Scientific |
| 8 | UGT1A1 | Hs02511055 | Thermo Fisher Scientific |
| 9 | NRF2 (NFE2L2) | Hs00975961 | Thermo Fisher Scientific |
| 10 | GPX4 | Hs00989766 | Thermo Fisher Scientific |
| 11 | CAR (NR1IH) | Hs00901571 | Thermo Fisher Scientific |
| 12 | MDR1 (ABCB1) | Hs00184491 | Thermo Fisher Scientific |
| 13 | MDR3 (ABCB4) | Hs00983957 | Thermo Fisher Scientific |
| 14 | MRP2 (ABCC2) | Hs00166123 | Thermo Fisher Scientific |
| 15 | BSEP (ABCB11) | Hs00184824 | Thermo Fisher Scientific |
| 16 | NTCP (SLC10A1) | Hs00161820 | Thermo Fisher Scientific |
| 17 | ACTB | Hs01060665 | Thermo Fisher Scientific |
| 18 | CTNNB1 | Hs00355045 | Thermo Fisher Scientific |
| 19 | IL1a | Hs00174092 | Thermo Fisher Scientific |
| 20 | TNF | Hs00174128 | Thermo Fisher Scientific |
| 21 | MYC | Hs0015340 | Thermo Fisher Scientific |
